# Change in the Pattern of Tuft-Level Wear in Wide-Head Manual Toothbrushes After Use: A Prospective Cohort Pilot Study

**DOI:** 10.7759/cureus.90658

**Published:** 2025-08-21

**Authors:** Yoshino Kaneyasu, Hideo Shigeishi, Yoshie Niitani, Toshinobu Takemoto, Masaru Sugiyama, Kouji Ohta

**Affiliations:** 1 Department of Public Oral Health, Program of Oral Health Sciences, Graduate School of Biomedical and Health Sciences, Hiroshima University, Hiroshima, JPN; 2 Department of Oral Health Management, Program of Oral Health Sciences, Graduate School of Biomedical and Health Sciences, Hiroshima University, Hiroshima, JPN; 3 Department of Oral Health Sciences, Faculty of Health Care Sciences, Takarazuka University of Medical and Health Care, Takarazuka, JPN

**Keywords:** bristle abrasion, bristle splaying, manual toothbrush, scanning electron microscopy (sem), toothbrush physical properties

## Abstract

Introduction: Changes in the physical properties of manual toothbrush bristles are key indicators of toothbrush effectiveness. While wide-head-type manual toothbrushes have recently gained popularity in Japan, limited research has examined how their bristle properties change over time. This study aimed to investigate time-dependent changes in the overall and individual tuft bristle characteristics of wide-head-type toothbrushes, compared with conventional compact-head-type toothbrushes.

Methods: Sixteen participants from Hiroshima University were initially enrolled. After excluding three individuals who withdrew for personal reasons, 13 participants completed the study. Toothbrushes were collected at baseline (T0), after one month (T1), and after two months (T2), with new toothbrushes provided at each time point. In total, 18 wide-head-type and 21 compact-head-type toothbrushes were analyzed. Seven tuft locations were assessed in the wide-head type, and eight in the compact-head type. Bristle splaying and abrasion were measured using a digital microscope and scanning electron microscopy (SEM). For overall bristle splaying, 864 tufts in total were assessed for the wide-head type (48 tufts per toothbrush) and 357 tufts in total for the compact-head type (17 tufts per toothbrush). For individual tuft bristle splaying and abrasion, 126 tufts in total were analyzed for the wide-head type (seven tufts per toothbrush) and 168 tufts for the compact-head type (eight tufts per toothbrush).

Results: No significant changes were observed in overall bristle splaying over the two-month period for the wide-head-type toothbrush. The wide-head-type toothbrush had fewer spread tufts than the compact-head-type. During the study period, the total abrasion score per tuft was significantly lower for the wide-head-type toothbrush than for the compact-head-type. Additionally, significant bristle splaying and abrasion were observed only in the rightmost middle region tuft of the wide-head type.

Conclusions: Wide-head-type toothbrushes were less susceptible to tuft splaying and abrasion. The number of spread tufts influenced overall brush deformation. Variations in tuft location may differentially affect the rate of bristle wear.

## Introduction

Daily toothbrushing is important not only for maintaining and promoting oral health, such as preventing gingivitis and periodontitis, but also for supporting general health [1-3]. Accordingly, various manual toothbrushes have been continuously improved to develop more effective tools for oral hygiene [4]. However, manual toothbrushes have a limited lifespan and must be replaced regularly to maintain their effectiveness [5]. Key indicators of toothbrush effectiveness and the need for replacement include changes in the physical properties of the bristles, such as splaying, bending, and abrasion [5-9]. As a result, several methods have been developed to investigate bristle spreading and abrasion over time with continued use [9-11].

Notably, not only the entire bristle head but also each tuft comes into contact with localized, minute regions of the oral cavity, such as the cervical areas of teeth, interproximal spaces, and the gingival margin. Previous studies have reported that changes in the physical properties of bristles may vary depending on the tuft location [8,12]. Based on these findings, it is important to investigate tuft-level alterations in physical properties when assessing the efficacy of manual toothbrushes.

The bristle material plays a crucial role in determining these physical properties and, by extension, the overall effectiveness of a toothbrush [13]. Common materials include nylon and saturated polyester resin [13]. Although nylon has traditionally been used for toothbrush bristles [14,15], its relatively low water resistance leads to reduced durability over time [13]. In contrast, saturated polyester resin is more water-resistant and stable under wet conditions, making it more durable and less prone to splaying [13].

In Japan, wide-head-type manual toothbrushes equipped with super-tapered bristles made of durable, low-water-absorbing saturated polyester resin have recently gained popularity [8,16]. The development of manual toothbrushes requires innovative designs that can accommodate variations in brushing technique and duration [14,17,18]. Wide-head-type toothbrushes can cover a larger area of the tooth surface and help offset individual differences in brushing habits. Consequently, these brushes have been reported to be useful for both individuals who can brush their own teeth and those who cannot [16]. These considerations suggest potential advantages of wide-head-type toothbrushes, although their efficacy still requires further empirical validation. To date, few studies have examined changes in the physical properties of bristles, particularly at the tuft level, in wide-head-type manual toothbrushes.

Therefore, this study aimed to quantitatively assess changes in splaying and abrasion at the tuft level in wide-head manual toothbrushes after baseline, as well as one and two months of routine use. In addition, this study sought to provide information for future manual toothbrush design.

## Materials and methods

Participants

**Figure 1:**
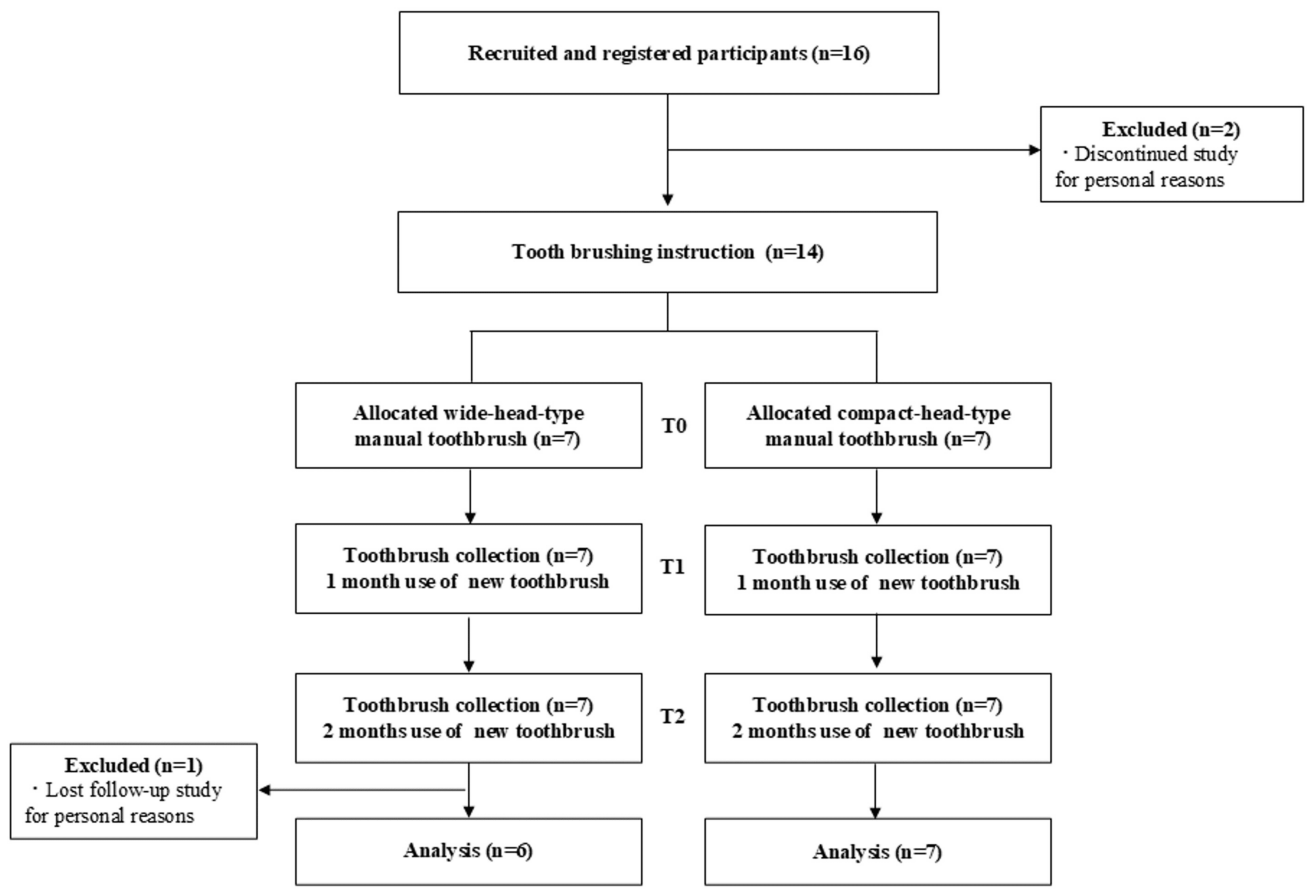
Flow diagram illustrating participant inclusion in the study

This prospective pilot study was conducted at Hiroshima University. Participation was voluntary, and 16 staff members were recruited between September 2021 and September 2022. After excluding three participants, 13 were included in the final analysis (Figure 1).

The study was approved by the Ethics Committee of Hiroshima University in June 2021 (approval number: E2021-2497). All participants signed informed consent forms. The study adhered to the Strengthening the Reporting of Observational Studies in Epidemiology (STROBE) statement [19].

The inclusion criteria were as follows: (a) age ≥ 18 years but <40 years, and (b) at least 18 remaining teeth, excluding third molars. “Remaining teeth” refers to the number of natural teeth currently present in the oral cavity, excluding implants and dentures. The exclusion criteria encompassed the following: (a) undergoing extensive prosthetic treatment (e.g., crown restorations or cavity treatment), (b) presence of orthodontic appliances, (c) pregnancy, (d) antibiotic use within the past three months, and (e) presence of severe systemic diseases.

Participants were non-randomly assigned to either the wide-head-type or compact-head-type manual toothbrush group, as predetermined by the researchers, and used the assigned toothbrushes for one or two months, respectively. Before the study began, all participants received toothbrushing instructions from a dental hygienist, accompanied by a leaflet. The leaflet provided guidance on the scrubbing method, to be performed twice daily for three minutes each time, the use of non-abrasive toothpaste, and the avoidance of other oral hygiene aids. The participants were also instructed to brush their teeth in small, continuous movements, one or two teeth at a time. The three-minute brushing time is sufficient for an adult to brush their entire dentition [20]. Using a non-abrasive toothpaste can minimize the influence of external factors, such as ablativity, on bristle wear patterns, thereby focusing our analysis on the differences between the tufts based on the brush heads themselves. Participants were also instructed to apply an appropriate toothbrushing pressure (150-200 g) using a toothbrushing pressure-measuring device (Komatsu Co., Ltd., Saitama, Japan) (Appendices). An appropriate tooth brushing pressure of 150-200 g can be confirmed by a meter and a melody sound. If the pressure exceeds 200 g, a buzzer will sound as a warning to the participant.

Based on these procedures, participants’ brushing techniques were standardized as much as possible throughout the study period, following the protocol of our previous studies [6,20]. Participants’ compliance with the brushing protocol was monitored daily using a calendar-type checklist. Toothbrushes were collected at baseline (T0), after one month of use (T1), and after two months of use (T2). The collected manual toothbrushes were numbered and blinded to the assessors so that they did not know which toothbrushes had been used for which period. The two-month usage period was based on previous reports indicating that the effectiveness of saturated polyester resin bristles (e.g., polybutylene terephthalate (PBT)) declines after two months [6].

Characteristics of wide- and compact-head-type manual toothbrushes

The toothbrushes used in this study were Systema-type brushes (LION Corp., Tokyo, Japan). Their characteristics are summarized in Table 1. The wide- and compact-head types differed in head width, vertical length, number of tufts, and number of bristles per tuft, while all other features were identical.

**Table 1:** Characteristics of the wide-head-type and compact-head-type manual toothbrushes ^a^From each of the wide- and compact-head types, three unused toothbrushes were selected, and the number of bristle filaments per tuft was counted.

Variables	Wide-head-type (DENT.EX systema genki)	Compact-head-type (Systema 44M)
Wide head size (mm)	14.4	8.1
Vertical head size (mm)	24.5	18.5
Bristle length (mm)	12	12
Bristle diameter (mm)	0.19	0.19
Bristle stiffness	Normal	Normal
Bristle material	Saturated polyester resin	Saturated polyester resin
Shape type of bristle tip	Super-tapered type	Super-tapered type
Tip thickness in the head (mm)	4.5	4.5
Number of bristles per tuft^a^	40-48	56-66
Total number of tufts transplanted in the head	48	17

Measurement of bristle splaying (entire and each tuft) using digital software

**Figure 2:**
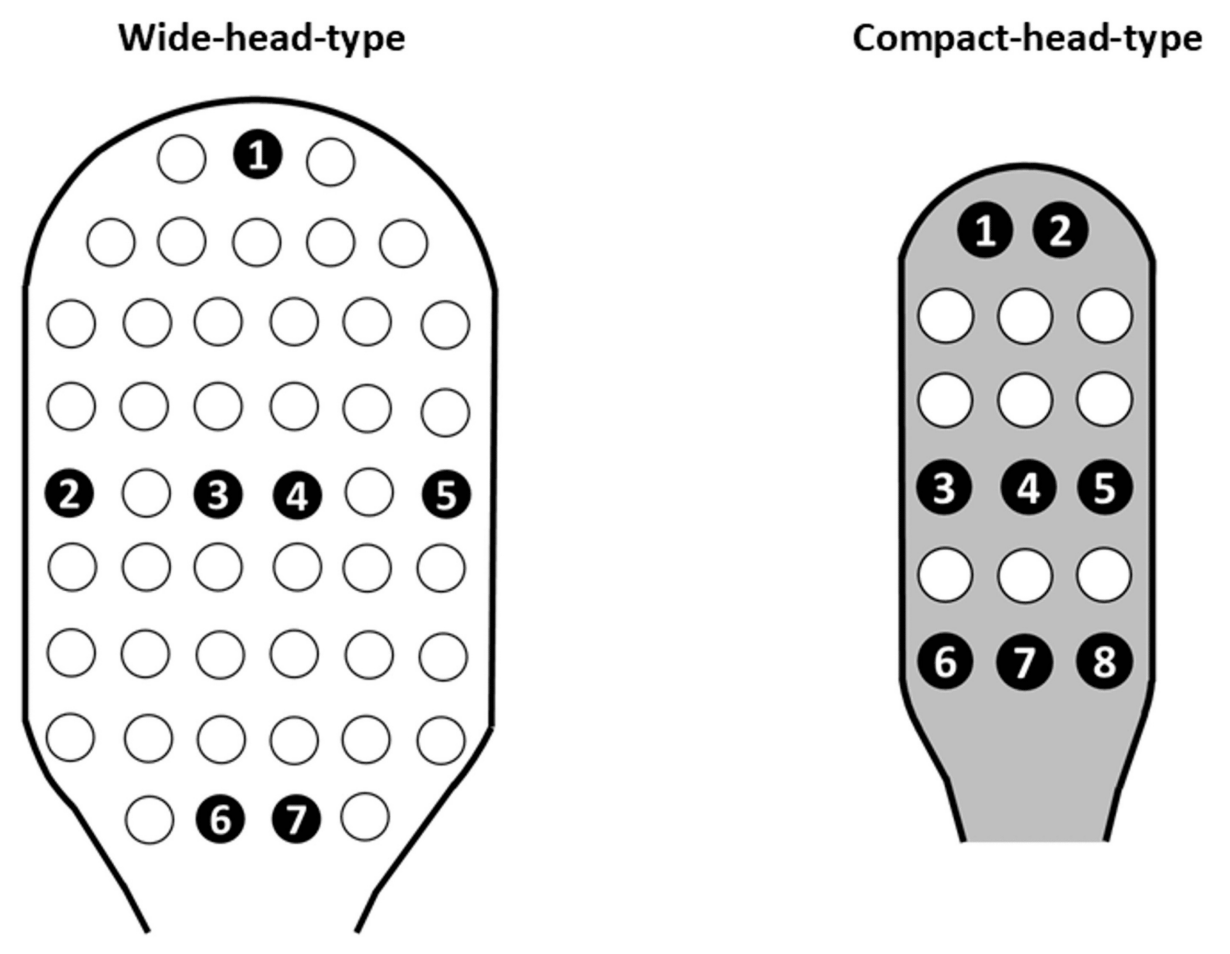
Illustration of the bristle splaying and abrasion measurement areas for each tuft

Each wide-head-type toothbrush had 48 tufts, and 18 such brushes were analyzed, totaling 864 tufts. Each compact-head-type toothbrush had 17 tufts, and 21 brushes were analyzed, totaling 357 tufts. Additionally, seven tuft locations on the wide-head type and eight on the compact-head type were evaluated on each collected toothbrush (Figure 2).

**Figure 3:**
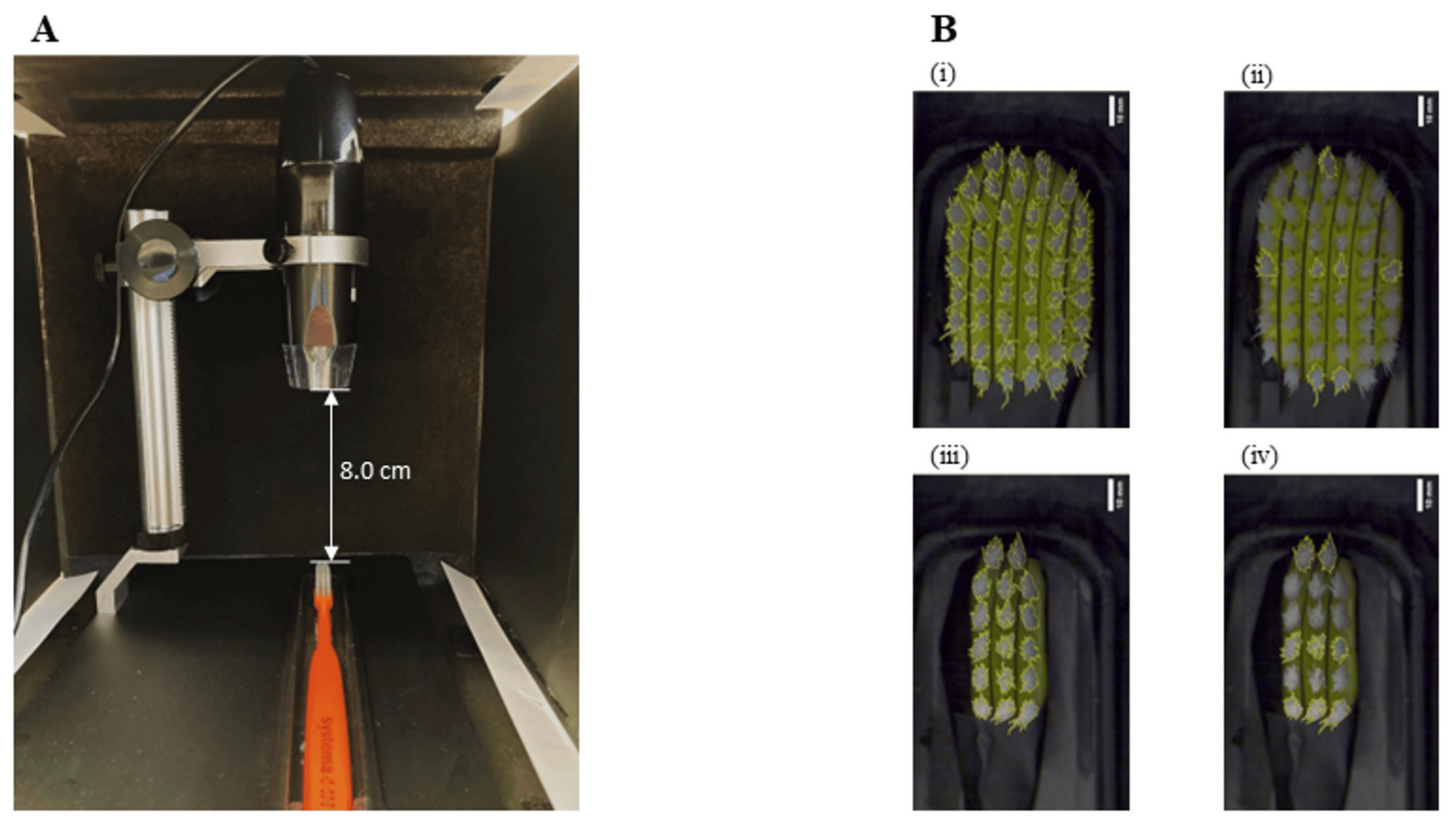
Measurement of overall and tuft-level bristle splaying (A) Device setup for measuring the splaying of the entire and individual tuft bristles. The distance from the bristle plane to the digital microscope lens was set at 8 cm. (B) Location of measurements for bristle splaying: (i) tuft locations in the wide-head type for measuring entire bristle splaying, (ii) seven tuft locations in the wide-head type for measuring individual tuft bristle splaying, (iii) tuft locations in the compact-head type for measuring entire bristle splaying, and (iv) eight tuft locations in the compact-head type for measuring individual tuft bristle splaying.

To accurately capture the vertical top view of each brush head, a digital microscope (3R-MSUSB401, 3R SOLUTION CORP., Fukuoka, Japan) equipped with a light-shielding box to block ambient light and a black horizontal stand was used. The brush head was placed on the stand, and the distance from the bristle plane to the digital microscope camera was fixed at 8 cm (Figure 3A). Images (640 × 480 pixels) were acquired, calibrated at 18.2 pixels/mm, and analyzed using NIH ImageJ software to measure each tuft’s area (Figure 3B). This method allowed the evaluation of a greater number of bristle bundles than our previous study [12].

Each toothbrush was analyzed three times (T0, T1, and T2), and the median of the three values was used. Reliability was assessed using the coefficient of variation (CV), calculated as (standard deviation/mean) × 100. The CV values of the surface area were 1.5% at T0, 1.4% at T1, and 1.4% at T2. According to de Ruiter et al., CV values ≤ 5% are considered highly reliable, 5.1%-10% moderately reliable, and >10% unacceptable [21]. Thus, the CV values in this study indicate high reliability.

Measurement of bristle abrasion using scanning electron microscopy

Bristle abrasion was assessed using scanning electron microscopy (SEM) (JSM-7200F; JEOL Ltd., Tokyo, Japan). Bristles were coated with platinum using a sputtering device (JFC-3000FC; JEOL Ltd., Tokyo, Japan) and observed under SEM at an accelerating voltage of 1 KV and magnifications of 30× and 100×. Images were captured from directly above and at a 70° angle, with a working distance of 12 mm. This method followed the protocol from a prior study [12]. Abrasion was measured at seven tuft locations for the wide-head type and eight for the compact-head type (Figure 2).

**Figure 4:**
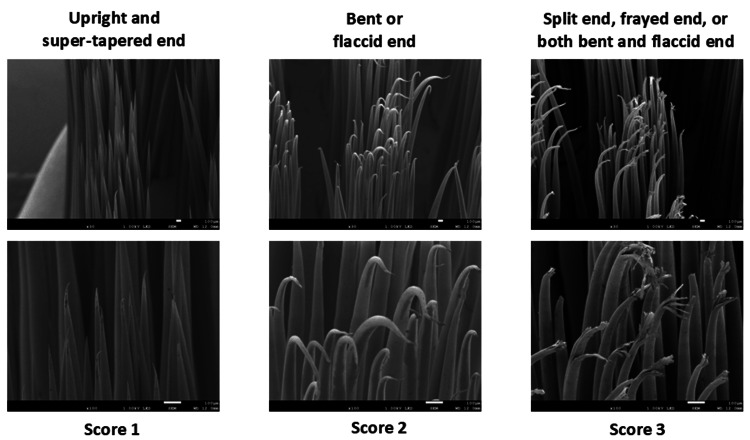
SEM-based classification of bristle tip abrasion Score 1: upright and super-tapered end, score 2: bent or flaccid end, score 3: split end, frayed end, or both bent and flaccid end SEM: scanning electron microscopy

The degree of bristle abrasion was rated on a 3-point scale based on the shape of the bristle tips observed in SEM images: (a) score 1: upright and super-tapered end, (b) score 2: bent or flaccid end, and (c) score 3: split end, frayed end, or both bent and flaccid ends (Figure 4).

These classifications were adapted from previous studies [22]. The abrasion score for each tuft was calculated by multiplying the number of bristles in each score category by the respective score and summing the results. The total score per toothbrush was then divided by the number of tufts evaluated (seven for wide-head and eight for compact-head types) to obtain the average abrasion score per tuft.

Statistical analysis

Descriptive analyses of all variables were expressed as medians with interquartile ranges (IQRs). The Mann-Whitney U test and Fisher’s exact test were used to evaluate significant differences in participant characteristics, bristle splaying, and abrasion between the wide-head-type and compact-head-type toothbrush groups. Continuous data on bristle splaying area and abrasion at T0, T1, and T2, as well as for each tuft, were analyzed using the Steel-Dwass test. All statistical analyses were performed using JMP Pro software version 18.0.1 (SAS Institute Inc., Cary, NC). A significance level of p < 0.05 was considered statistically significant.

To assess whether the sample size was adequate, a post hoc power analysis was conducted using G*Power version 3.1.9.4 (Heinrich Heine University Düsseldorf, Düsseldorf, Germany) [23], based on the effect size observed in the comparison tests. The statistical power of the Wilcoxon Mann-Whitney test (for two independent groups) was calculated using the total sample size, assuming an effect size of 0.5 and a significance level of 0.05. The resulting power exceeded 0.80, indicating that the study had sufficient statistical power to detect meaningful differences, in accordance with Cohen’s guidelines [24].

## Results

Participants of this study

The characteristics of the participants are presented in Table 2. A small number of participants, two before the brushing instructions and one from the wide-head-type toothbrush group, discontinued the intervention for personal reasons unrelated to the study (Figure 1). No significant differences were observed between the two groups in terms of age, gender, or number of remaining teeth. During the study, the participants’ brushing compliance was satisfactory, with wide-head type scores of 95.97% (90.73%, 98.79%) at T1 and 100% (100%, 100%) at T2, and compact-head type scores of 87.1% (83.87%, 100%) at T1 and 94.35% (85.48%, 100%) at T2.

**Table 2:** Characteristics of participants using wide-head-type and compact-head-type manual toothbrushes ^a^Mann-Whitney U test ^b^Fisher’s exact test p < 0.05 was considered statistically significant. ^†^“Remaining teeth” indicates the number of natural teeth currently present in the oral cavity, excluding implants and dentures. IQR: interquartile range

Variables	Wide-head type (n = 6)	Compact-head type (n = 7)	U-value	p-value
Age, median (IQR)	29.5 (27.8-31.3)	29 (29-33)	16.5	0.561^a^
Gender, number (%)				
Male	5 (83.3)	2 (28.6)	-	0.103^b^
Female	1 (16.7)	5 (21.5)	-	
Number of remaining teeth^†^, median (IQR)	28 (27-28.3)	28 (28-28)	21.0	> 0.99^a^

Changes in bristle splaying of the entire and individual tufts in wide- and compact-head-type manual toothbrushes

The bristle-splaying areas are summarized in Table 3. No significant changes were observed in the overall surface area of bristle splaying at T0, T1, or T2 for the wide-head-type toothbrush. In contrast, the surface area of the compact-head-type toothbrushes was significantly greater at T2 compared to T0 (p < 0.05).

**Table 3:** Changes in bristle splaying of the entire brush and individual tufts in wide-head-type and compact-head-type manual toothbrushes after use *Steel-Dwass test used for within-group comparisons p < 0.05 was considered statistically significant. *p < 0.05 The entire bristle-splaying area of the compact-head-type toothbrushes was significantly greater at T2 than at T0 (p < 0.05). For the wide-head-type toothbrush, tuft 5 showed significantly greater splaying at both T1 and T2 compared to T0 (p < 0.05). In the compact-head-type toothbrush, significant splaying was observed in tufts 3 and 6 at T1 and T2 compared to T0 (p < 0.05), and in tufts 5 and 8 at T1 compared to T0 (p < 0.05). T0: baseline, T1: one month, T2: two months, IQR: interquartile range

	Bristle splaying area (mm²)	T0 median (IQR)	T1 median (IQR)	T2 median (IQR)
Wide-head type	Entire	115.95 (114.64-117.80)	118.98 (110.25-134.68)	130.82 (116.68-144.62)
	Tuft 1	3.02 (2.63-3.48)	2.84 (2.48-3.28)	3.16 (2.89-3.68)
	Tuft 2	3.36 (2.97-3.69)	2.96 (2.53-3.93)	2.46 (2.35-3.77)
	Tuft 3	2.22 (1.92-2.44)	1.92 (1.79-2.49)	2.28 (1.91-2.70)
	Tuft 4	1.87 (1.78-2.25)	1.93 (1.60-2.24)	2.33 (1.94-2.83)
	Tuft 5	1.81 (1.67-2.01)	2.69^*^ (2.06-2.86)	2.35^*^ (2.33-2.93)
	Tuft 6	2.79 (2.42-2.86)	2.87 (2.42-3.47)	3.08 (2.31-3.48)
	Tuft 7	2.42 (2.27-2.63)	2.79 (2.34-3.70)	2.68 (2.37-3.22)
Compact-head type	Entire	40.52 (39.89-45.42)	49.89 (44.54-59.16)	53.99^*^ (52.31-68.69)
	Tuft 1	3.51 (3.31-3.67)	3.56 (3.07-3.72)	4.27 (3.80-4.47)
	Tuft 2	3.36 (3.11-3.67)	3.34 (3.18-3.42)	3.82 (3.63-4.30)
	Tuft 3	2.36 (2.29-2.45)	3.07^*^ (2.46-3.37)	3.52^*^ (2.81-4.00)
	Tuft 4	2.21 (2.16-2.38)	2.84 (2.11-3.38)	3.01 (2.27-3.38)
	Tuft 5	2.13 (1.85-2.37)	2.65^*^ (2.41-3.23)	2.93 (2.60-3.54)
	Tuft 6	2.07 (2.05-2.14)	2.56^*^ (2.43-3.36)	3.30^*^ (2.41-4.02)
	Tuft 7	2.11 (1.90-2.40)	3.05 (2.27-3.60)	2.93^*^ (2.52-4.05)
	Tuft 8	2.26 (2.17-2.40)	3.37^*^ (3.01-4.23)	3.12 (2.75-4.16)

Regarding changes in the surface area of each tuft’s bristle splaying, tuft 5 in the wide-head-type toothbrush showed significantly greater values at both T1 and T2 compared to T0 (p < 0.05). For the compact-head-type toothbrush, significant increases were observed for tufts 3 and 6 at T1 and T2 compared to T0 (p < 0.05), and for tufts 5 and 8 at T1 compared to T0 (p < 0.05).

Significant differences in the overall bristle splaying area were also observed between the wide- and compact-head-type toothbrushes at T0, T1, and T2 (p < 0.01).

Changes in bristle abrasion of individual tufts in wide- and compact-head-type manual toothbrushes

**Figure 5:**
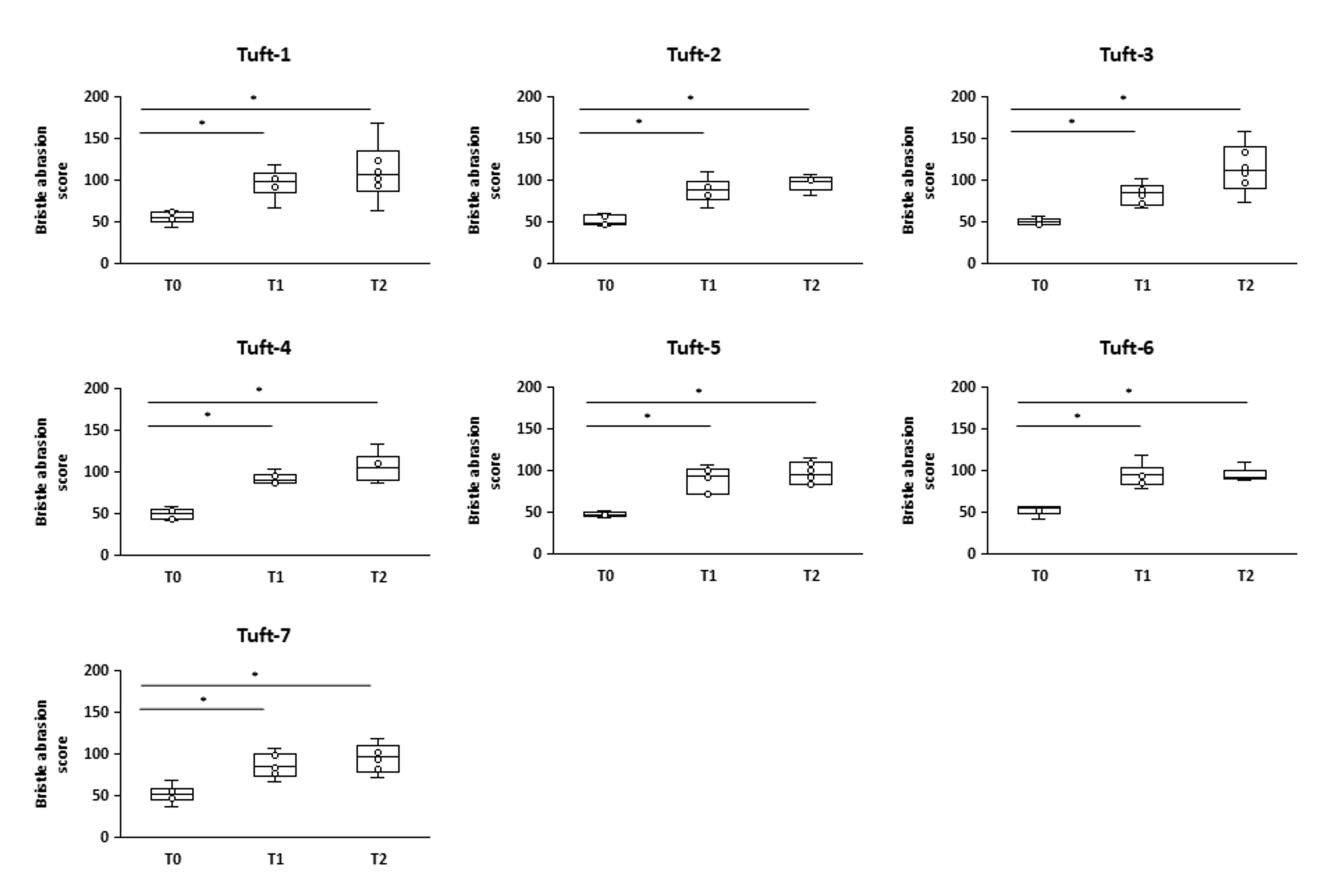
Changes in bristle abrasion scores for each tuft of the wide-head-type toothbrush at T0, T1, and T2 *Steel-Dwass test p < 0.05 was considered statistically significant. *p < 0.05 T0: baseline, T1: one month, T2: two months

The abrasion scores for all tufts in the wide-head-type toothbrushes showed significant increases at T1 and T2 compared to T0 (p < 0.05) (Figure 5).

**Figure 6:**
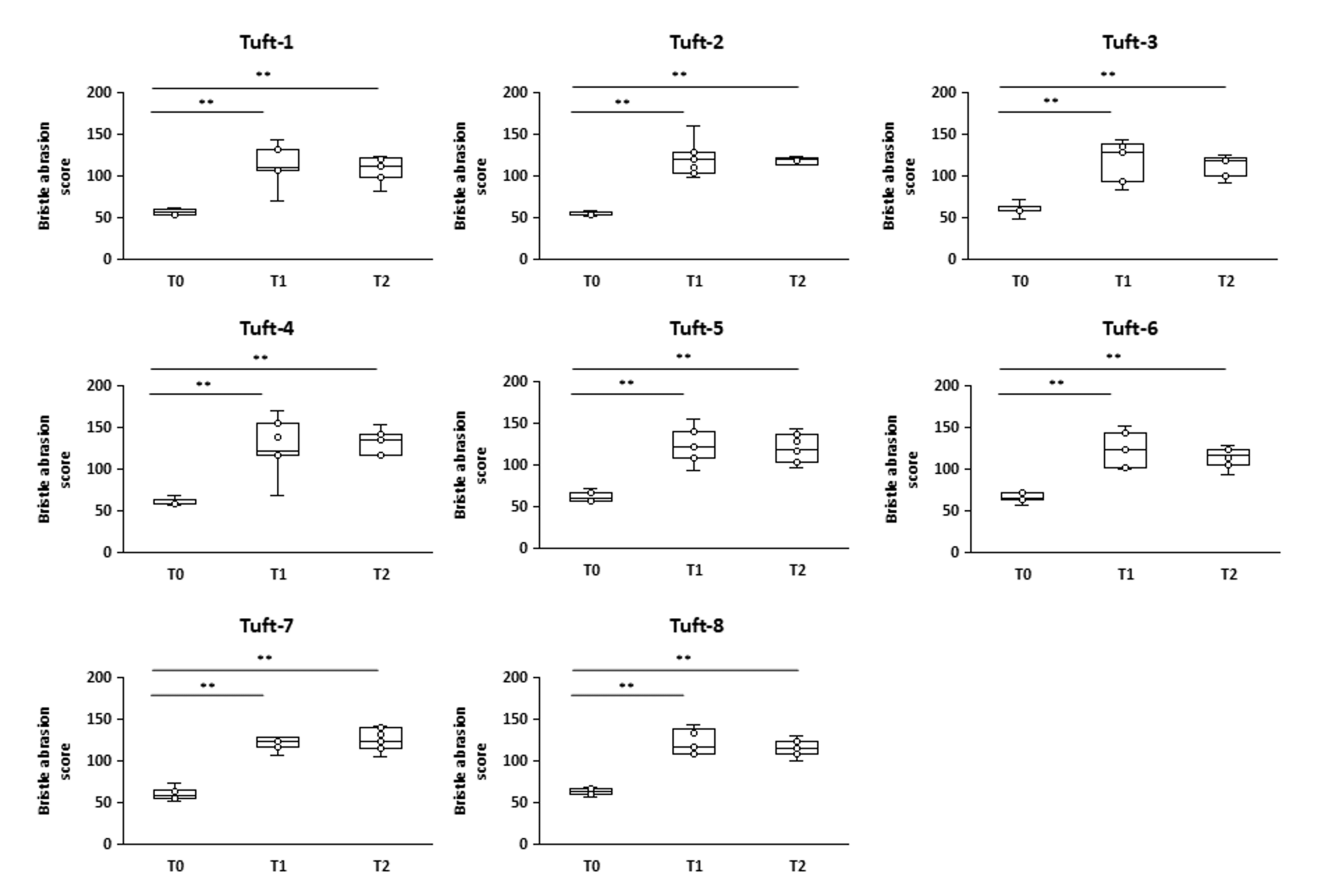
Changes in bristle abrasion scores for each tuft of the compact-head-type toothbrush at T0, T1, and T2 *Steel-Dwass test p < 0.05 was considered statistically significant. **p < 0.01 T0: baseline, T1: one month, T2: two months

Similarly, the compact-head-type toothbrushes exhibited significantly increased abrasion scores in all tufts at T1 and T2 compared to T0 (p < 0.01) (Figure 6).

**Figure 7:**
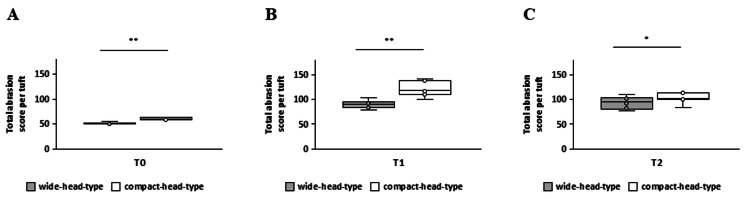
Comparison of total tuft bristle abrasion scores per tuft between wide- and compact-head-type toothbrushes at each time point A: T0, B: T1, C: T2 *Mann-Whitney U test p < 0.05 was considered statistically significant *p < 0.05, **p < 0.01 T0: baseline, T1: one month, T2: two months

Significant differences were also observed in the total abrasion scores per tuft between the wide- and compact-head-type toothbrushes at T0, T1, and T2 (p < 0.05) (Figures 7A, 7B, 7C, respectively).

## Discussion

This study investigated both the overall and individual bristle tuft splaying and abrasion of wide-head-type manual toothbrushes featuring saturated polyester resin with super-tapered tips, compared with conventional compact-head-type toothbrushes.

After two months of use, bristle splaying was minimal in the wide-head-type toothbrush but significantly more pronounced in the compact-head-type. Additionally, the number of significantly splayed tufts was higher in the compact-head-type group. Rawls et al. suggested that tuft placement and bristle interaction distances influence toothbrush performance [9]. Furthermore, de Bragança et al. reported that higher bristle volumes and thinner filament diameters are associated with increased abrasion indices in vitro [25]. This in vitro study reported that among seven types of manual toothbrushes with different bristles, the CS5460, Carbon, and SlimSoft toothbrushes, which are ultrasoft with numerous bristles, showed higher spreading and wear rates than the other toothbrushes [25]. In addition, these toothbrushes might have indicated a higher visual wear rate in a short time [25]. Since both toothbrush types used in this study had filaments of identical diameter, it is possible that the greater number of tufts in the wide-head type reduced abrasion despite having a larger total bristle mass. This possibility raises the hypothesis that the bristles of wide-head-type toothbrushes physically interact and reinforce each other, thereby suppressing the spreading of individual bristles and the overall bristles. Rawls et al. reported that a closely spaced tuft arrangement caused contact between bristles, resulting in friction [9]. The magnitude of bristle-to-bristle friction might increase the contact area between bristles, supporting our hypothesis that bristles support each other. This is a novel finding regarding the physical behavior of wide-head-type manual toothbrush bristles.

At the tuft level, significant bristle splaying was observed only in the rightmost middle region of the wide-head-type brush, whereas the compact-head-type brush exhibited significant splaying in the rightmost, leftmost, and lower tuft regions. A prior study divided the brush head into five regions (two central and three marginal) to assess one or seven bristles on each tuft to standardize tuft-based assessments [26,27]. To assess the tuft bristles on the toothbrush head comprehensively, five locations were identified for evaluating the center and edges of the head [26,27]. Our evaluation, based on over seven tuft locations and investigation of all bristles on each tuft, allowed for a more detailed characterization of physical property changes across both central and marginal tuft regions.

Both toothbrush types demonstrated significant bristle abrasion after one and two months of use. Interestingly, our previous SEM-based study found no signs of abrasion after three months in toothbrushes made from the same saturated polyester resin but featuring tapered (not super-tapered) tips [12]. These findings suggest that super-tapered tips, although made from durable resin, may be more susceptible to abrasion. Nonetheless, within this study, the wide-head-type toothbrush exhibited less wear than the compact-head-type.

Various methods have been employed to evaluate bristle abrasion, including stereomicroscopy and SEM [22,27,28]. SEM, in particular, offers detailed insights into bristle tip degradation [27]. However, most prior studies focused on nylon bristles, with limited research on saturated polyester resin bristles [22]. To our knowledge, few studies have evaluated tuft-level bristle tip wear after use [27,28]. The SEM-based evaluation index developed in this study enabled a comprehensive assessment of all bristles within a tuft, allowing the detection of even minor abrasion, despite the durability of the saturated polyester resin bristles.

Based on the findings of this study, the number, position of tufts on the head, and shape of the bristle tips should be considered when designing and developing new toothbrushes. The pattern of wear change at the tuft level may also be considered for assessing toothbrush replacement duration in clinical settings.

This study had some limitations. First, we could not assess how changes in physical properties affected oral hygiene outcomes, such as plaque removal, due to concerns about droplet transmission during the COVID-19 pandemic. Most studies evaluating oral hygiene to assess the effectiveness of toothbrushes have a three-month study period [8,10]. Following previous studies [6], our study was limited to two months; future studies should investigate the long-term bristle wear patterns of three months or longer and acquire sufficient evidence to strengthen the correlation with real-world oral health effects. Second, user comfort and ease of use were not evaluated, despite the comfort and convenience of using a toothbrush representing essential information for users [29]. Positive perceptions and satisfaction with oral care products, such as toothbrushes, may contribute to maintaining and improving long-term oral health, as well as demonstrating the efficacy of toothbrushes [29,30]. Future studies should assess user satisfaction and ergonomics for wide-head-type toothbrushes. Third, this study did not investigate ingredients in toothpaste other than abrasives. Participants were instructed to use a non-abrasive toothpaste that did not contain any active abrasive agents. This strategy was used intentionally to minimize the potential influence of toothpaste abrasiveness on the bristle splaying and abrasion, allowing us to focus on the effects of the tufts based on different brush head types. However, further research into the relationship between bristle properties and oral hygiene status will require the standardization or description of the detailed composition of toothpastes. Lastly, although the observed statistical power was high, the small sample size and post hoc power calculation limit generalizability; the limitation remains significant and warrants cautious interpretation. Furthermore, randomization of participants was not performed, although no significant difference in participants’ characteristics was observed between the two groups in this study. Future research with high-quality studies and larger sample sizes should be conducted.

## Conclusions

The wide-head-type manual toothbrush did not exhibit significant bristle splaying after two months of use and demonstrated lower susceptibility to tuft-level splaying and abrasion compared to the compact-head-type toothbrush. Additionally, the number of splayed tufts may contribute to overall bristle spread. Bristle degradation rates appear to vary depending on tuft location. These findings suggest that manufacturers should consider information about changes in the physical properties of tuft bristles when designing new toothbrushes.

## Figures and Tables

**Figure 8:**
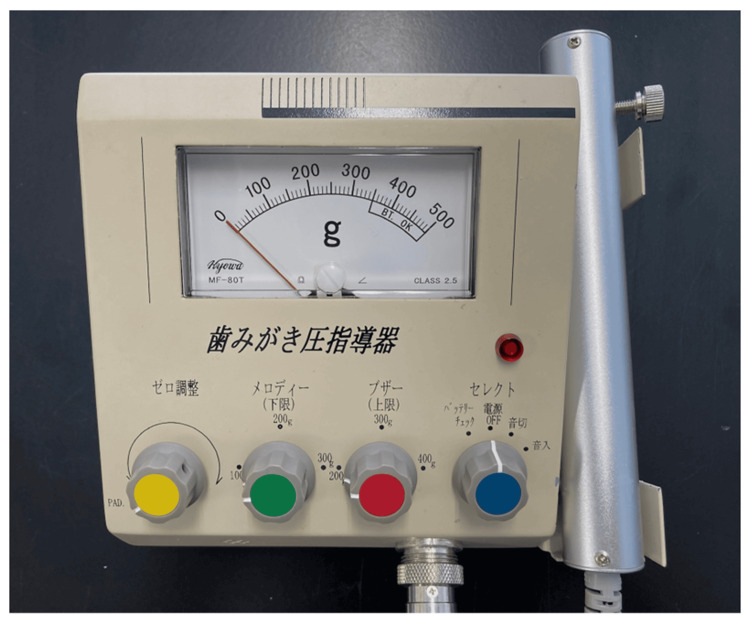
Toothbrushing pressure-measuring device (Komatsu Co., Ltd., Saitama, Japan)

## Appendices

The toothbrushing pressure-measuring device used in the present study is shown in Figure 8.
